# Genome-Wide Identification and Expression Analysis of the *WSD* Gene Family in Wheat

**DOI:** 10.3390/genes17030353

**Published:** 2026-03-23

**Authors:** Chang Liu, Zelin Niu, Huaihai Yu, Bingyan Gu, Yifei Jia, Denglei Xie, Rongna Wang

**Affiliations:** 1 School of Health and Pharmaceutical Sciences, Zibo Polytechnic University, Zibo 255300, China; liuchang_bio@foxmail.com (C.L.);; 2 State Key Laboratory of North China Crop Improvement and Regulation, Key Laboratory of Hebei Province for Plant Physiology and Molecular Pathology, College of Life Sciences, Hebei Agricultural University, Baoding 071001, China; niuzl200012@163.com (Z.N.);

**Keywords:** WSD, wheat, gene family, phylogeny, lamina joint, plant architecture

## Abstract

Background: Wax synthase/diacylglycerol acyltransferases (WS/DGATs), often referred to as WSD proteins, represent a class of key enzymes that catalyze the biosynthesis of wax esters in plants and other organisms. However, the WSD gene family in wheat (*Triticum aestivum*) has not been systematically characterized. Methods: A comprehensive genome-wide identification and bioinformatic characterization of the WSD gene family were conducted in wheat, followed by an analysis of chromosomal locations, gene structures, conserved motifs, phylogenetic relationships, expression profiles, and cis-element predictions. Results: In this study, a total of 43 *TaWSDs* were identified through genome-wide analysis in wheat. All identified TaWSD members exhibit highly conserved structural features and contain the core catalytic motif HHXXXDG. Phylogenetic analysis of WSD proteins from 63 species revealed that WSDs in Triticeae, including wheat, were mainly clustered into four distinct clades. Furthermore, sequence divergence among TaWSDs from different clades was primarily localized to the N-terminal region. Notably, expression profile analysis demonstrated that *TaWSD* genes display organ-specific expression patterns in wheat. Among them, 12 *TaWSDs* showed the highest expression levels in the leaf lamina joint, implying their potential involvement in the regulation of leaf angle formation. Additionally, 27 transcription factors were computationally predicted as putative regulators of *TaWSDs*, although their exact roles require further experimental confirmation. Conclusions: Our findings provide novel insights into the biological functions of the wheat *WSD* gene family and offer new perspectives for elucidating their molecular mechanisms underlying plant architecture regulation.

## 1. Introduction

During their evolutionary adaptation to terrestrial life, plants have evolved a hydrophobic cuticle covering their aerial surfaces, which is made of cutin and epicuticular waxes and acts as the primary barrier against various environmental stresses [1]. The plant cuticle fulfills multiple critical biological functions: it reduces non-stomatal water loss [2,3], reflects excessive ultraviolet radiation [4], and blocks the invasion of pathogens [5,6]. Thus, the biosynthesis of cutin and epicuticular waxes is essential for plants to cope with a wide range of abiotic and biotic challenges. Beyond its protective functions, the cuticle also participates in the specification of organ boundaries and the establishment of cell polarity. For example, numerous *Arabidopsis thaliana* mutants with defective cuticle biosynthesis commonly exhibit organ fusion and reduced plant stature [7,8,9,10]. Ma et al. (2024) found that the cuticle is indispensable for skotomorphogenesis and plays a key role in organ initiation and development [11]. In terms of plant reproduction, the cuticle serves as an important structural component for anther development, pollen formation, and the regulation of male fertility [12,13,14].

Wax esters are a class of neutral lipids and components of plant cuticular waxes, which are primarily synthesized through the esterification of long- or very-long-chain aliphatic alcohols and acids [15]. These compounds impart strong hydrophobicity and enhance the barrier properties of the cuticle [16,17]. The wax ester synthase/diacylglycerol acyltransferase (WS/DGAT) family constitutes a class of enzymes that catalyze wax ester biosynthesis [18]. WS/DGAT (WSD) proteins function as bifunctional O-acyltransferases with both wax ester synthase and diacylglycerol acyltransferase activities [19], and they possess conserved domains in both N- and C-terminal halves [20]. The first plant *WSD* gene was identified and characterized in petunia [21]. The *A. thaliana* genome encodes 11 WSD family members (*AtWSD1*-*AtWSD11*), but only *AtWSD1* has been experimentally validated for its biological function [15]. Li et al. (2008) [15] demonstrated that AtWSD1 is localized to the endoplasmic reticulum, displays high wax synthase activity, and is specifically expressed in floral tissues, the apical regions of stems, and leaves. The WSD protein family is widely distributed across the plant kingdom, although the copy number of *WSD* genes varies significantly among different plant species [22]. Phylogenetic analyses have shown that WSD proteins from monocots and eudicots form distinct evolutionary clades [22]. Notably, in comparison to other DGAT enzymes, the ADP1 protein from *Acinetobacter* sp. Falls within a shared clade with plant WSDs, suggesting that the divergence of this family occurred before the emergence of land plants. In most species, WSD genes contain seven exons, and their encoded WSD proteins harbor six conserved motifs [22]. Moreover, functional studies have demonstrated that WSD proteins typically possess a highly conserved catalytic motif HHXXXDG, which is critical for their enzymatic activity [17,23].

Wheat is one of the most important cereal crops worldwide and plays a pivotal role in global food security [24]. The cuticle exerts a significant positive effect on stabilizing and improving the yield potential of wheat [25,26]. Previous studies have shown that cuticular wax composition in wheat is governed by the acyl elongation—reduction—decarbonylation pathway and the β-diketone pathway [27]. Despite these advances, the knowledge of genes involved in wax ester biosynthesis in wheat remains limited. Additionally, most studies focus on the role of WSD in plant responses to abiotic stresses [28,29,30]. However, in this study, we found that several *TaWSD* genes exhibited expression patterns closely associated with leaf lamina joint (LJ) development. The lamina joint is specialized mechanical tissue specific to the Poaceae family, located at the junction between the leaf blade and leaf sheath, where it determines the leaf angle [31,32]. As plant architecture-related traits, leaf angle has been recognized as a key agronomic trait that determines light interception, photosynthetic efficiency, and yield potential under high-density planting conditions [24,33,34]. Previous studies have reported that lipid metabolism and cell wall organization are involved in the LJ developmental process [32], but their potential roles in modulating LJ development and leaf angle formation remain largely unexplored.

To date, genome-wide identification and expression profiling of the WSD gene family have only been reported in a limited number of plant species, such as sunflower [17] and apple [35]. In addition, the transcriptional regulatory mechanism of WSDs remains unclear, as only two transcription factors, WRI4 and MYB94, have been reported to positively regulate WSD1 in *A. thaliana* [36,37]. Thus, we conducted a comprehensive genome-wide identification and bioinformatic characterization of the WSD gene family in wheat, analyzing chromosomal locations, gene structures, conserved motifs, and phylogenetic relationships. Subsequently, gene expression profiling was carried out, followed by the prediction of cis-regulatory elements and transcriptional regulators. Wax esters are a class of lipids with poorly understood functions, and their roles in plant architecture remain largely unknown. In this study, the findings not only expand our understanding of the biological functions of *WSD* genes in plants but also provide novel insights into the regulation of cuticular wax biosynthesis during wheat organ development, a process potentially linked to plant architecture establishment.

## 2. Materials and Methods

### 2.1. Identification of WSD Genes in Wheat and Other Species

This study used the genome and annotation of bread wheat (*T. aestivum*, cv. Chinese Spring) from the Ensembl Plants database. *A. thaliana* WSD genes (*AtWSDs*) were retrieved from Li et al. [15]. We first performed BLASTP v2.12.0 searches of the AtWSD protein sequences against the wheat proteome with an e-value cutoff of ≤1 × 10^−5^. Given that all AtWSDs contain two conserved domains—the wax ester synthase/diacylglycerol acyltransferase catalytic domain (PF03007) and the WS/DGAT C-terminal domain (PF06974)—we further used hmmscan (HMMER software v3.3.2) to identify wheat proteins harboring both domains. A wheat gene was classified as a WSD family member (TaWSD) only if its encoded protein exhibited homology to AtWSDs and contained both conserved domains. The same pipeline was applied to identify WSD family members in other plant species.

### 2.2. Chromosomal Distribution and Physicochemical Property Analysis of TaWSDs

Gene coordinates of *TaWSDs* were obtained from the genome annotation, and their chromosomal distribution was visualized using R v4.5.2. Protein sequences of TaWSDs were used to compute molecular weight, isoelectric point (pI), and hydrophobicity with the Peptides package in R. Subcellular localization of TaWSD proteins was further predicted using the online tool DeepLoc-2.1.

### 2.3. Phylogenetic and Synteny Analysis of the WSD Gene Family

Phylogenetic trees for WSDs in *T. aestivum*, *Triticum turgidum*, and *Aegilops tauschii* and WSDs in *Triticum urartu*, *Aegilops speltoides*, rice, maize, and *A. thaliana* were constructed as follows: protein sequences were aligned using MAFFT v7.525, trimmed with TrimAl v1.5, and phylogenies inferred with FastTree v2.1.11. Trees were visualized using the R package ggtree.

The tree of WSDs comprising 63 species (Table S1), was generated using the same alignment and trimming pipeline (MAFFT and TrimAl), but reconstructed with IQ-TREE2 v2.3.4 and visualized via iTOL v7. Clades were retained only if they met both criteria of bootstrap support ≥ 70 and ≥5 genes per clade, yielding 12 major clades.

For intra- and inter-genomic synteny analyses in wheat, pairwise protein sequences were first aligned using BLASTP with the following thresholds: e-value ≤ 1 × 10^−10^, identity ≥ 30%, and coverage ≥ 50%. The resulting hits were used as input for MCScanX to identify syntenic blocks, which were visualized with the R package circlize. Gene duplication modes (e.g., tandem, segmental, dispersed, proximal) were classified using MCScanX’s duplicate_gene_classifier module based on BLASTP alignment results of wheat protein sequences and GFF3 annotation files.

### 2.4. Gene and Protein Structure Analysis

The transcript structures of TaWSDs (including CDS, 5’UTR, and 3’UTR regions) were extracted from the genome annotation files (GFF3 format). We utilized R scripts to parse these coordinates and visualize the exon-intron organization.

The positions of conserved domains in their protein sequences were identified using InterProScan. Additionally, conserved motifs in the protein of TaWSDs were analyzed with MEME v4.11.2 using the following parameters: -mod anr -nmotifs 9 -minw 40 -maxw 250. Subsequently, the protein sequences were rescanned with FIMO v4.11.2 (*p*-value < 1 × 10^−9^) based on the MEME results to map the positions of these conserved motifs within the proteins. The three-dimensional (3D) structure of the TaWSD proteins was predicted using AlphaFold3 (https://alphafoldserver.com/). The selection of TaWSD proteins for 3D structure visualization was based on the PTM values derived from the predicted results. Additionally, the 3D structure was visualized using Pymol software v3.0.

### 2.5. Transcriptome Profiling Analysis

In this study, we constructed expression profiles using transcriptome data from 201 wheat samples. Raw sequencing reads (Table S2) were downloaded from the NCBI SRA database and converted to FASTQ format using SRA Toolkit v3.1.0. The reads were then preprocessed with trim-galore v0.6.10, which automatically detected and removed adapter sequences and low-quality bases. After quality control, the cleaned reads were aligned to the reference genome using HISAT2 v2.2.1, and strand specificity was evaluated with the RSeQC package v5.0.2. Transcript assembly and quantification of gene expression levels across samples were performed using StringTie v2.2.2.

For downstream expression analysis, only genes with an FPKM ≥ 0 in at least one sample were retained. Wheat samples were first clustered based on global expression patterns using hierarchical clustering (hclust, method = “average”). Then we employed the cluster.stats function from the R package fpc to evaluate clustering validity. Based on the Dunn2 Index, we initially identified k = 37 as the optimal number of clusters. Since some of these 37 clusters contained mixed tissue types, we further subdivided these specific clusters based on tissue identity, resulting in 43 sample groups. Among these, six groups had a sample size of less than three, which was insufficient for robust statistical analysis. Consequently, we merged these small groups with their phylogenetically nearest neighbors based on the phylogenetic tree topology, yielding a final set of 38 sample groups. The PCA analyses within the tissue samples were performed and visualized by R package FactoMineR and factoextra. Then, mean expression values of TaWSDs were then calculated for each group. The FPKM values were the transformed using log2(FPKM+1) to stabilize variance. Subsequently, to visually highlight the relative expression ranking of each gene across samples, we applied Min-Max normalization. Each gene’s expression profile was independently scaled to a range of 0 to 1, where 0 represents the minimum expression level and 1 represents the maximum expression level observed across all samples for that specific gene. Finally, a heatmap of expression trends, integrated with the WSD phylogenetic tree, was generated using R package ggplot2 and ggtree.

### 2.6. Quantitative Real-Time PCR (qRT-PCR) Analysis

The wheat (*T. aestivum* L.) seedlings were grown in a greenhouse with 16 h light (12,000 lux)/8 h dark cycles and a constant temperature of 25 °C. For qRT-PCR sample collection, three stages were defined: Stage 1 (S1), when the lamina joint just emerged (4 days after seed germination); Stage 2 (S2), 8 days after seed germination; and Stage 3 (S3), 12 days after seed germination, when the leaf angle had fully opened. To ensure sufficient sample quantity, each sample was prepared by pooling and grinding tissues from multiple plants, followed by thorough homogenization (n ≥ 10). Eastep^®^ Super Total RNA Extraction Kit (Promega Beijing Biotech Co., Ltd., Beijing, China) was used to isolate total RNA, and reverse transcription reactions were performed using the two step PrimeScript RT Reagent Kit with gDNA Eraser (Takara Bio Inc., Dalian, China) by following the protocol. RT-qPCR experiments were conducted using TB Green^®^ Premix Ex Taq™II (Tli RNaseH Plus) (Takara Bio Inc., Dalian, China). The *GAPDH* gene served as the control (Table S3), and relative expression changes were calculated using the 2^−ΔΔCt^ method [38]. The specific primers of candidate genes for qRT-PCR were shown in Table S3. For RT-qPCR analysis, three technical replicates were performed for each of the three biological replicates in each group. RT-qPCR data were analyzed using R v4.5.2. The significance test for differences among different treatments was performed via Tukey’s HSD post hoc test implemented in the agricolae package of R.

### 2.7. Cis-Regulatory Element and Potential Regulatory Factor Analysis

Cis-regulatory elements in the promoters of TaWSDs associated with hormone response, stress response, light responsiveness, and developmental processes were predicted using the PlantCARE database. The results were visualized using the R package ComplexHeatmap.

Transcription factors (TFs) in wheat were predicted by the web-tool of PlantTFDB database. To identify transcription factors (TFs) potentially regulating TaWSD genes, we performed global correlation analysis and intra-tissue correlation analysis. Pearson correlation coefficients (PCCs) were calculated between the expression profiles of TaWSDs and individual TFs; TFs showing a PCC ≥ 0.9 across both analytical dimensions were designated as candidate regulators. These candidates were hierarchically clustered based on their correlation with TaWSD expression using hclust function (method = “average”) in R. The optimal number of clusters was determined using the fpc package, resulting in 6 distinct clusters. Both the correlation matrix and expression profiles were visualized by R package ComplexHeatmap.

Furthermore, potential transcription factor binding sites (TFBSs) in promoter regions of TaWSDs were predicted by scanning against the JASPAR_plant_core database (2024 release) using FIMO. From the resulting hits, we filtered for motifs corresponding to homologs of our candidate TFs (identified based on highest sequence similarity using BLASTP, parameters: -evalue 0.00001 -max_target_seqs 1). We then analyzed the distribution of these specific motifs within the TaWSD promoter regions and identified candidate motif–TaWSD pairs to support the predicted regulatory relationships. The occurrence count of these selected motifs in each TaWSD promoter was quantified, and the resulting matrix was visualized using the R package ComplexHeatmap, while their specific positions within the promoter regions were plotted using custom R scripts.

## 3. Results

### 3.1. Identification of TaWSDs and Physicochemical Property Analysis

A total of 43 WSD family members were identified in the bread wheat (*T. aestivum*) genome, which were designated TaWSDs and named according to their chromosomal positions and sequence homology (Table 1; Figure S1). The amino acid sequences ranged from 382 to 538 in length, with predicted molecular weights of 42.24–59.96 kDa and theoretical isoelectric points (pI) spanning 6.09 to 9.92. Notably, 36 members (83.72%) had a pI greater than 7, indicating that most wheat WSD proteins are basic in nature. Additionally, more than half of the TaWSD proteins were predicted to be hydrophilic, while 15 were characterized as hydrophobic. Furthermore, all TaWSDs are predicted to target the endoplasmic reticulum (ER), and the majority (36 members) contain transmembrane domains (Table S4), which is consistent with the subcellular localization characteristics of WSD proteins reported in other plant species [15,35].

### 3.2. Phylogenetic and Synteny Analysis of TaWSDs

**Figure 1:**
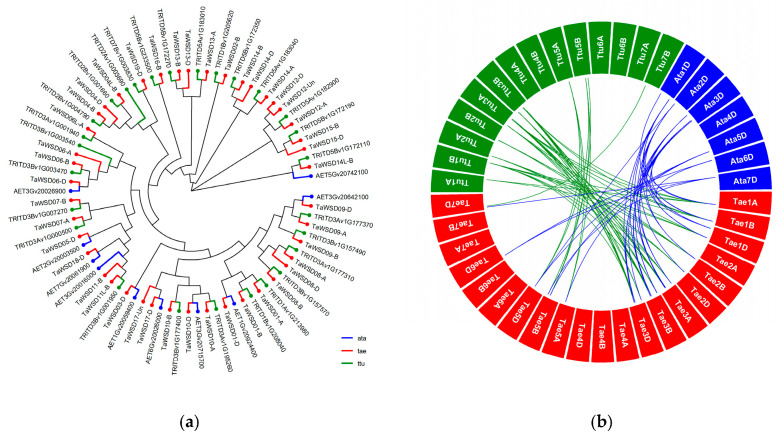
Phylogenetic and syntenic analyses of WSDs in *T. aestivum*, *T. turgidum*, and *A. tauschii*. (**a**) Phylogenetic tree of WSD genes. Red, green, and blue tips represent genes from *T. aestivum*, *T. turgidum* (ttu), and *A. tauschii* (ata), respectively. (**b**) Collinear gene pairs identified by syntenic analysis. Green links connect gene pairs between *T. aestivum* and *T. turgidum*; blue links connect those between *T. aestivum* and *A. tauschii*.

Our study found that the number of WSD genes in wheat (43) is far greater than that in rice (4) and maize (2), indicating an expansion of the WSD family in wheat. Phylogenetic analysis showed that the most *TaWSDs* have orthologous genes in the tetraploid progenitor *Triticum turgidum* (AABB) and the D-genome donor *Aegilops tauschii* (Figure 1a). Interspecific synteny analysis revealed that 29 *TaWSDs* are syntenic with genes in *T. turgidum* or *A. tauschii*, corresponding to 65 and 44 collinear gene pairs, respectively (Figure 1b; Table S5). Among them, 23 pairs were identified as putative orthologs. Within the wheat genome, we also identified 40 intraspecific collinear gene pairs involving 26 *TaWSDs* (Table S5). Furthermore, we classified the *TaWSDs* according to their gene duplication modes, including 28 segmental, 11 tandem, 3 dispersed, and 1 proximal duplication (Figure S2A). Notably, *TaWSDs* without interspecific collinear genes were exclusively derived from tandem duplications events (Figure S2B). Chromosomal localization analysis revealed distinct distribution patterns of these tandemly duplicated genes (Figure S1): one subset is clustered within homologous regions of chromosomes 3A, 3B, and 3D, while another forms significant tandem gene clusters on chromosomes 5A, 5B, and 5D.

**Figure 2:**
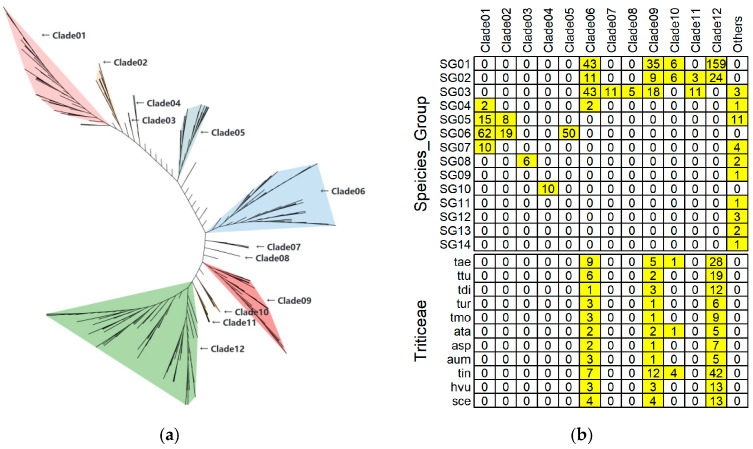
Phylogenetic analyses of WSD genes in 63 species. (**a**) Phylogenetic tree of WSD genes. (**b**) Distribution of WSD genes across 14 taxonomic groups (SG01-SG14): Triticeae (SG01), other Pooideae (SG02), other Poaceae (not Pooideae, SG03), other Poales (not Poaceae, SG04), other Monocots (not Poales, SG05), Eudicots (SG06), Angiosperms (not Monocots or Eudicots, SG07), Lycopodiopsida (Vascular plants, SG08), Polypodiopsida (Vascular plants, SG09), Bryophyta & Marchantiophyta (Embryous plants, SG10), Streptophyta (SG11), Chlorophyte (SG12), Rhodophyta (SG13) and Bacteria (outgroup, SG14). The Triticeae group includes *T. aestivum* (tae), *Triticum turgidum* (ttu), *Triticum dicoccoides* (tdi), *Triticum urartu* (tur), *Triticum monococcum* (tmo), *A. tauschii* (ata), *Aegilops speltoides* (asp), *Aegilops umbellulata* (aum), *Thinopyrum intermedium* (tin), *Hordeum vulgare* (hvu), *Secale cereale* (sce).

In addition, the diploid relatives of bread wheat, *Triticum urartu* and *Aegilops speltoides*, each contain 10 *WSD* genes, a number still higher than that in rice or maize. These results revealed that the *WSD* genes form distinct yet relatively conserved clades (Figure S3), indicating that this gene family underwent an early expansion within the Triticeae lineage. To further explore whether wheat and its closely related species have evolved lineage-specific WSD subfamilies, we conducted a comprehensive genome-wide identification and phylogenetic analysis of the *WSD* gene family across 63 species (Table S6 and Figure 2a). The selected taxa span a broad evolutionary range, including 35 species in Monocots, 13 species in Eudicots, 3 species in other angiosperms, 1 species in Polypodiopsida, 2 species in Lycopodiopsida, 3 species belonging to Bryophyta and Marchantiophyta, 1 species in Streptophyta, 3 species in Chlorophyta, 1 species in Rhodophyta, and 1 species in Bacterial (served as the outgroup). Phylogenetic analysis revealed that WSD members from Triticeae species predominantly cluster into four clades, namely Clade06, Clade09, Clade10, and Clade12 (Figure 2b). Notably, Clade10 and Clade12 display a high degree of specificity for the Pooideae in SG1 and SG2 and lack any WSD members from non-Pooideae lineages (Figure 2b). Specifically, Clade12 contains 183 *WSD* genes from 13 Pooideae species, while Clade10 contains only 12 *WSD* genes from 4 Pooideae species. In *T. aestivum* (tae), 1 and 28 WSD members belong to Clade10 and Clade12, respectively (Figure 2b). Additionally, Clade06 and Clade09 show relative specificity to Poales and Poaceae, respectively. Clade06 comprises 99 WSD genes from 28 Poales species, and Clade09 includes 62 WSD genes from 25 Poaceae species. In *T. aestivum* (tae), 9 and 5 WSD members are assigned to Clade06 and Clade09, respectively. Interestingly, two WSD genes from *Joinvillea ascendens* (the sister group to Poaceae within Poales [39,40]) in Clade06 were identified, suggesting that Clade06 originated early during Poales diversification. In contrast, Clade10 and Clade12 were exclusively restricted to Pooideae members including Triticeae, *Avena sativa*, and *Lolium perenne*. By integrating this finding with published divergence times [41], we infer that Clade10 and Clade12 originated prior to the split of core Pooideae tribes (Aveneae, Triticeae, Lolieae) approximately 28.2 million years ago.

### 3.3. Gene and Protein Structure of TaWSDs

**Figure 3:**
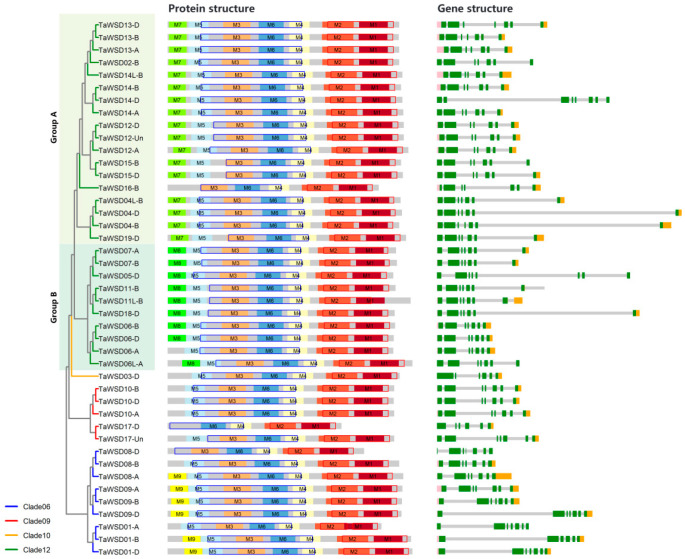
Gene and Protein Structure of TaWSDs. In the protein structure diagram, M1–M9 represent conserved motifs of the protein. Transparent squares with blue borders indicate PF03007, while those with red borders represent PF06974. In the gene structure diagram, green represents CDS, pink represents 5’ UTR, and orange represents 3’ UTR.

The gene lengths of *TaWSDs* ranged from 2.89 kb to 13.09 kb, of which 39 members (90% of the total) are shorter than 10 kb. All *TaWSD* genes exceeding 10 kb belonged to Clade 12. In terms of exon-intron structure, the number of exons of *TaWSDs* was highly conserved (Figure 3). Among them, 38 members contained 7 exons, 3 had 6 exons, and 1 member possessed 8 or 9 exons. Additionally, 33 *TaWSDs* members are characterized by the presence of both annotated 5′ UTRs and 3′ UTRs, implying potential post-transcriptional regulation and spatiotemporal expression mechanism that calls for further investigation.

InterProScan analysis confirmed that all TaWSDs harbor two conserved domains, including the catalytic WS/DGAT domain and the WS/DGAT C-terminal domain. Moreover, the results of the conserved motif analysis (Figure 3) showed that the majority of TaWSD proteins shared six core motifs (M1–M6), while only one member lacked M3, and three lacked M5. Some TaWSD members additionally contained specific motifs M7–M9. Motif M7 is predominantly present in a subclade of Clade 12 (designated Group A, Figure 3), which includes 17 members. Motif M8 is exclusively found in another subclade of Clade 12 (designated Group B, Figure 3), comprising 9 members. Motif M9 is primarily distributed in Clade 06, with 6 members harboring this motif. These findings indicated that TaWSD proteins within the same phylogenetic clade exhibited highly conserved motif compositions, while the motif differences among different clades might be associated with the functional diversification during evolution.

Notably, the conserved catalytic motif HHXXXDG, which is essential for WSD enzymatic activity, is embedded within Motif M3 (Figure S4). With the exception of TaWSD17-D, all other family members were predicted to contain a canonical M3 motif. Further sequence alignment analysis revealed that the N-terminal region of TaWSD17-D still showed high sequence similarity (maximum identity ≥ 70%) to the M3 motif and retained the core HHXXXDG segment (Figure S4). The failure of MEME to identify a complete M3 motif in TaWSD17-D was likely due to its truncated N-terminal region. Additionally, reduced conservation of the HHXXXDG segment was observed in TaWSD18-D, TaWSD11-B, and TaWSD11L-B, where the glycine (G) at the C-terminus of the motif was substituted with alanine (A) (Figure S4). Nevertheless, 3D structural modeling results suggested that this single amino acid substitution did not significantly alter the local conformational structure and this region still functioned as a β-strand-to-α-helix transition segment (Figure S5).

### 3.4. Expression Patterns of TaWSDs

**Figure 4:**
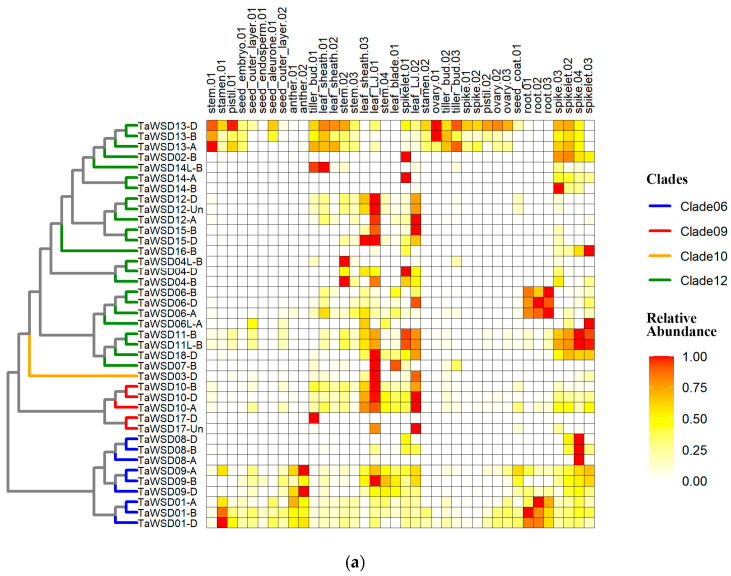

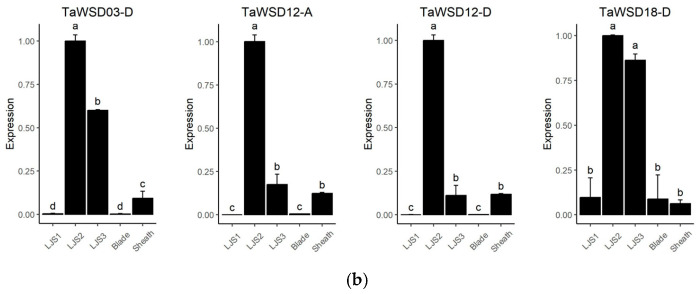
Expression profiles of *TaWSDs*. (**a**) Expression patterns of *TaWSDs* across different tissue groups. Only genes with a maximum FPKM ≥ 1 are shown. (**b**) Validation of 4 *TaWSD* genes with peak expression in leaf LJs by qRT-PCR. Each value represents the mean ± SD of three biological replicates. Different letters above the bars indicate significant differences at *p* < 0.05 using Tukey’s HSD post hoc test following one-way ANOVA.

We performed a comprehensive expression profiling analysis using transcriptomic data from 201 wheat samples representing 17 distinct tissue types (Table S2), which were divided into 38 distinct sample groups (Figure S6). Expression abundance analysis showed that *TaWSDs* were almost not expressed in the seed endosperm (maximum FPKM < 1) and exhibited generally low expression in other seed tissues, such as the seed outer layer, seed coat, embryo, and aleurone (maximum FPKM < 5), implying that the *TaWSD* gene family had little involvement in the developmental processes of seeds in wheat. Analysis of the expression atlas further revealed that four *TaWSDs* genes (*TaWSD05-D*, *TaWSD07-A*, *TaWSD14-D*, and *TaWSD19-D*) displayed consistently low expression across all tissue groups (maximum FPKM < 1), whereas the remaining *TaWSDs* were relatively highly expressed in at least one tissue group (Figure 4a), indicating functional divergence within the *TaWSD* gene family. For instance, within Clade 06, the *TaWSD08s* were preferentially expressed in spikes, and *TaWSD01s* were predominantly expressed in roots. The *TaWSD10s* within Clade 09 were mainly expressed in leaf LJs. Within Clade 12, most genes showed high expression levels in leaves and stems, with the exception of *TaWSD06s* and *TaWSD13s*. The *TaWSD06s* displayed predominant expression in roots, and the *TaWSD13s* were expressed ubiquitously across a range of vegetative and reproductive organs.

Notably, we found that the *TaWSD12s*, *TaWSD15s* and *TaWSD18* in Clade12, *TaWSD3* in Clade10, *TaWSD10s* and *TaWSD17-Un* in Clade09, *TaWSD09-B* in Clade06 exhibited peak expression in the leaf LJs, which are key mechanical organ regulating leaf angle in grasses. Furthermore, qRT-PCR results showed that *TaWSD03-D*, *TaWSD12-A*, *TaWSD12-D* and *TaWSD18-D* were all specifically highly expressed in the LJ tissue compared to leaf blade and sheath. Additionally, during LJ development, *TaWSD12-A* and *TaWSD12-D* expression peaked at the S2 stage, *TaWSD03-D* and *TaWSD18-D* showed higher expression at both S2 and S3 stages (Figure 4b). These results further validated the specific expression of these *TaWSD* genes in the leaf LJ and their potential association with LJ development and leaf angle formation.

### 3.5. Cis-Regulatory Elements and Potential Transcriptional Regulators of TaWSDs

**Figure 5:**
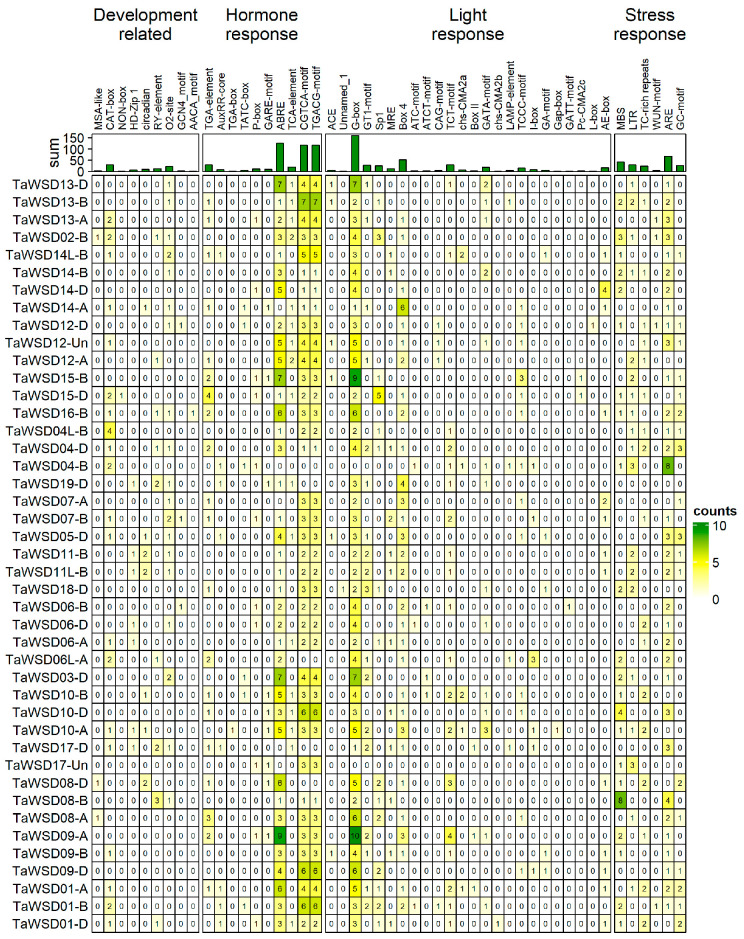
Cis-acting regulatory element of *TaWSDs* on promoter region.

To investigate potential transcriptional regulators of *TaWSDs*, cis-regulatory elements in their promoter regions were predicted using the PlantCARE database. Elements associated with hormone responses, stress responses, light responses, and developmental regulation were identified (Figure 5). The analysis revealed that over 80% of the *TaWSD* promoters harbored abscisic acid (ABA)-responsive ABRE elements, methyl jasmonate (MeJA)-responsive CGTCA-motif and TGACG-motif elements, and the light-responsive G-box element. Additionally, specific motifs were notably enriched in a few promoters. For instance, eight drought-responsive MBS elements were predicted in the *TaWSD08-B* promoter, and eight anaerobic stress-responsive ARE elements were identified in the *TaWSD04-B* promoter.

**Figure 6:**
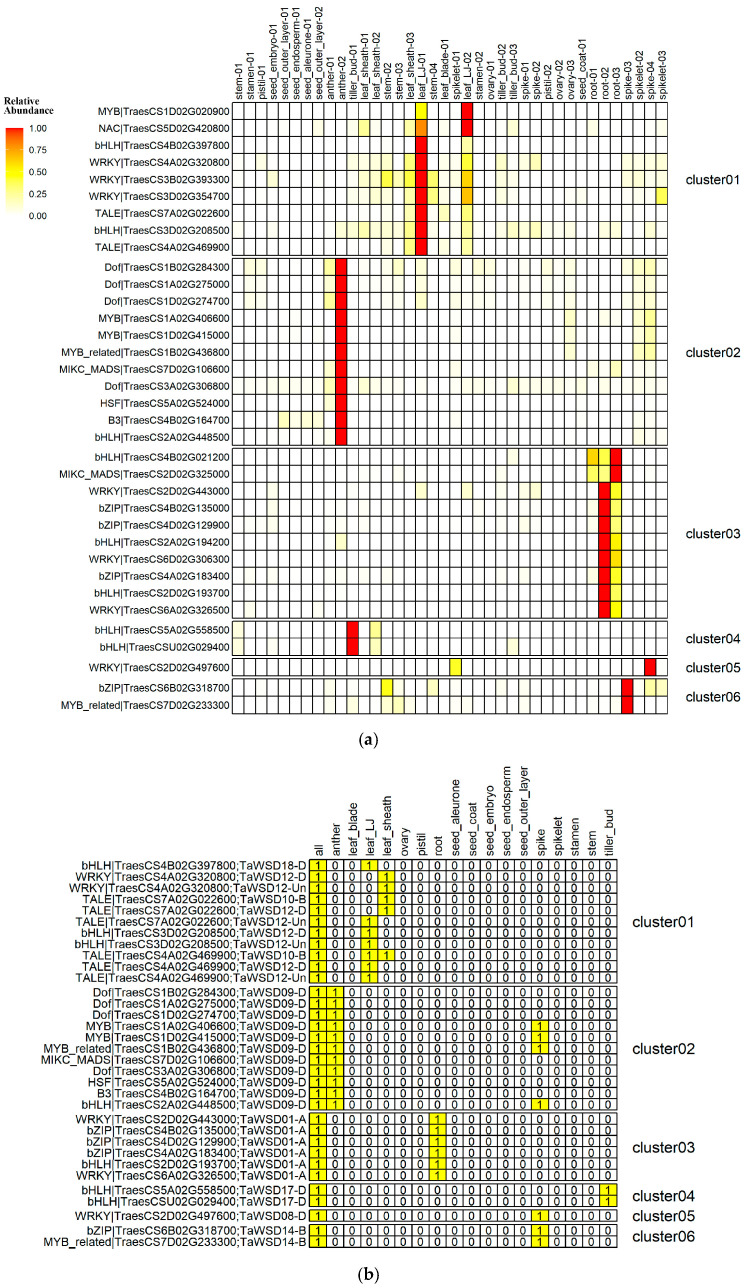
Predictive TFs regulating TaWSDs. (**a**) Expression patterns of 35 TFs identified in global correlation analysis. (**b**) Potential TF-target gene pairs identified in both global correlation analysis and intra-tissue correlation analysis (PCC ≥ 0.9).

Subsequently, we performed a co-expression analysis between 5878 wheat TFs annotated in PlantTFDB and the *TaWSDs* using available transcriptomic data. In the global correlation analysis, a total of 35 TFs were identified (Table S7). Based on the distribution of PCC values, these TFs were grouped into six clusters (Cluster01–06, Figure S7). Remarkably, each cluster of TFs exhibited a distinct tissue-specific expression patterns. For example, Cluster01 was highly expressed in leaf LJ, Cluster02 predominantly in anthers, Cluster03 specifically in roots, and Cluster04 primarily in tiller buds, while Clusters 05 and 06 showed dominant expression in spikes (Figure 6a). However, only 33 gene pairs (involving 27 candidate TFs) were retained after integrating positive correlations across tissue-specific samples (PCC ≥ 0.9; Figure 6b). Interestingly, the co-expression patterns of these retained pairs matched their known tissue specificities. For instance, *TraesCS4A02G469900*, *TraesCS4B02G397800*, and *TraesCS3D02G208500* were highly expressed in LJ tissues and showed significant positive correlations with specific *TaWSD* genes exclusively within the LJ sample set.

Additionally, among the 27 identified candidate TFs, 5 belonged to Cluster 01 and were assigned to bHLH, WRKY, and TALE families. Cluster 02 contained 11 TFs representing seven families (Dof, MYB, MYB_related, MIKC_MADS, HSF, B3, and bHLH), which were potentially associated with regulating *TaWSD09-D*. Six TFs from Cluster 03 belonged to four families (bHLH, MIKC_MADS, WRKY, and bZIP) and were putatively linked to *TaWSD01-A*. Two bHLH-family TFs from Cluster 04 were predicted to regulate *TaWSD17-D*, while a single WRKY-family TF from Cluster 05 might target *TaWSD08-D*. Finally, two TFs from Cluster 06, belonging to the bZIP and MYB_related families, were potentially involved in regulating *TaWSD14-B*. Collectively, the high consistency between these TF expression patterns and the organ-specific expression of *TaWSD* genes implied a potential regulatory relationship.

To further investigate potential regulatory relationships, we analyzed the distribution of predicted transcription factor binding sites (TFBS) in the promoter regions of the corresponding *TaWSD* genes (Table S8). The results showed that only four TFBS-target gene pairs were consistent with predictions based on positive expression correlations (Figure S8), including TraesCS4A02G469900 (TALE family) regulating *TaWSD10-B*, TraesCS4A02G320800 (WRKY family) regulating *TaWSD12-Un*, and TraesCSU02G029400 and TraesCS5A02G558500 (both bHLH family) regulating *TaWSD17-D*. Notably, all TFBS motifs in these regulatory pairs were located within 500 bp upstream of the transcription start site (TSS), providing strong support for these potential TF-target gene regulatory relationships (Figure S8).

## 4. Discussion

### 4.1. Lineage-Specific Expansion and Structural Conservation of the WSD Family in Wheat

In this study, we identified 43 *TaWSD* genes in wheat (*T. aestivum*), far exceeding the number found in rice and maize, suggesting a significant expansion of this gene family within the Triticeae lineage. Synteny analysis revealed that approximately 67% of *TaWSDs* were retained from ancestral progenitor genomes during allopolyploidization, indicating that polyploidy events contributed substantially to the expansion of this family. Moreover, the retention of similar cluster structures across all three subgenomes strongly suggests that most of the tandem duplication events likely occurred prior to the polyploidization of the Triticum genus (i.e., during the era of their diploid common ancestor) and were subsequently retained as intact genomic segments. These findings provide crucial spatiotemporal insights into the evolutionary history of the WSD gene family. Phylogenetic analysis across 63 species further supported a lineage-specific expansion pattern. Clade 06 was restricted to Poales, whereas Clades 10 and 12 were almost exclusively confined to Pooideae. Phylogenetic reconstruction across a broader taxonomic spectrum further illuminates the deep evolutionary trajectory of the WSD family, encompassing clades exclusive to eudicots (Clade 05), bryophytes (Clade 04) and unique algal lineages. The presence of WSD homologs in basal streptophyte algae such as Chara braunii, clustering within the land plant lineage, indicates that the core WSD architecture was established early in streptophyte evolution and has been maintained throughout terrestrial plant diversification. Structurally, all TaWSD proteins harbor the conserved catalytic WS/DGAT domain and C-terminal domain, with conserved motif compositions strictly aligned with phylogenetic clades. Clade-specific motifs (M7-M9) are localized to the N-terminal regions, while the C-terminal catalytic core remains highly conserved. This pattern is consistent with a common evolutionary strategy in which the N-terminus diversifies to mediate functional specialization, such as altered substrate specificity or protein interaction, while the C-terminus preserves essential enzymatic activity [42,43]. Although variations in the conserved HHXXXDG motif (e.g., G→A in TaWSD18-D, TaWSD11-B, TaWSD11L-B) were observed, 3D modeling predicted minimal conformational disruption, suggesting that these changes may not abolish catalytic function.

### 4.2. Organ-Specific Expression Links TaWSDs to Leaf Lamina Joint Development and Plant Architecture

Our multi-tissue expression atlas revealed that most *TaWSD* genes exhibited pronounced organ-specific patterns, with particularly striking enrichment in the leaf lamina joint (LJ). The LJ is a specialized mechanical tissue specific to the Poaceae family that determines the leaf angle [31,32,44], a key agronomic trait influencing plant architecture and grain yield [33,34]. Leaf LJ is a highly specialized tissue in which cell morphology and cell wall properties directly govern leaf support and movement [31,32,44]. Previous studies have revealed that WSDs played vital roles in plant drought tolerance [28,29], through decreasing cuticular permeability to water [30]. In addition, defects in cuticle composition have been reported to affect epidermis formation [45]. The LJ defines a critical developmental interface adjacent to organ boundaries at the junction of the leaf blade and sheath. In our study, twelve *TaWSDs* showed peak expression in LJ tissues, and qRT-PCR validated the LJ-specific expression of TaWSD03-D, TaWSD12-A, TaWSD12-D, and TaWSD18-D during LJ development. Additionally, *TaWSD* genes were highly expressed during the later stages (S2 and S3) of LJ development, suggesting that wax ester deposition is not essential for LJ initiation but rather for its developmental process. Therefore, we propose that wax esters contribute to boundary maintenance between the leaf blade and sheath by locally modulating their deposition pattern [46], thereby influencing epidermal development.

Moreover, it has been reported that leaf epicuticular waxes reflect a portion of incident light, facilitating its scattering into lower canopy layers [47] and enhancing community-level photosynthetic efficiency. However, under high irradiance, photoinhibition can damage the photosynthetic machinery [48]. The cuticle serves as a physical shield that limits UV penetration [49], acting in concert with epidermal UV-absorbing compounds to provide photoprotection. Although the LJ primarily functions to regulate leaf angle, wax ester accumulation in its cuticle likely contributes to balancing light utilization and stress resilience through reinforcement of cuticular barrier properties. Additionally, cuticle integrity is crucial for preserving the spatial distribution of local signaling molecules such as salicylic acid, which is vital for defense against biotic stressors [50]. Consequently, wax esters, the potentially key constituents of the LJ cuticle, may also bolster pathogen defense responses in this region [51]. In summary, the LJ-specific expression of *TaWSDs* suggests that wax metabolism contributes to LJ development and leaf angle formation, thereby expanding the functions of cuticle-associated genes beyond stress protection.

### 4.3. Transcriptional Regulation of the Wheat WSD Genes

In this study, we identified 27 transcription factors, whose expression positively correlated with that of *TaWSDs*, spanning 10 families including bHLH, WRKY, TALE, Dof, MYB, MYB_related, MIKC_MADS, HSF, B3 and bZIP. Although members of several TF families, including MYB and bHLH have been previously implicated in the regulation of cuticle wax biosynthesis (Table S9), only two TFs, WRI4 [36] and MYB94 [37], have been directly linked to *WSD* gene regulation to date. However, neither of these two known regulators emerged as co-expressed candidates in our analysis. For LJ tissues, 12 *TaWSD* genes were identified with specific high expression and 5 of 27 TFs were further pinpointed through co-expression analysis as their potential upstream regulators, mainly from bHLH, WRKY, and TALE families showing the strongest links to LJ-expressed *TaWSDs*. Intriguingly, many members of these TF families in rice and maize have been reported to be involed in LJ development and leaf angle formation (Table S10) through modulating hormone signaling [52,53,54] or genes involved in cell elongation and lignification [55]. Among them, the bHLH family is particularly well-documented, with several members positively regulating leaf angle, including OsILI1–8 [54,56], OsBIM1 [57], OsbHLH079 [58], OsbHLH92 [59], OsBC1 [60,61], ZmbHLH30 [55] and ZmbHLH155 [55], and others acting as negative regulators, including OsAIF1 [62], OsAIF2 [62], OsbHLH157 [56], OsbHLH158 [56], OsbHLH98 [63], ZmIBH1 [64]. In addition, several additional TFs have been identified as positive regulators of leaf angle, including OsWRKY53 [53], OsWRKY72 [65], OsWRKY108 [66], and ZmKN1 [67]. Notably, *TraesCS1D02G020900*, a candidate identified in global correlation analysis, is homologous to *OsMYB7* [68] and shows strong correlation with those of *TaWSD10-D*, *TaWSD12-A*, and *TaWSD15-B*, indicating the potential participant of these genes in LJ development. These results suggest a conserved transcriptional framework for leaf angle regulation in cereals.

## 5. Conclusions

In summary, this study provides a comprehensive analysis of the WSD gene family in wheat and explores the potential roles of its members in lamina joint development. Our findings provide a foundation for further deciphering the biological functions of WSD genes, and offer new insights and directions for elucidating the molecular mechanisms underlying leaf angle regulation in wheat. Looking ahead, we propose generating CRISPR/Cas9-edited and overexpression lines for specific *TaWSD* genes to facilitate targeted lipidomics and permeability assays.

## Figures and Tables

**Table 1:** Genome-wide identification of TaWSDs in wheat.

Gene Name	Gene ID	Genomic Position	AS	Amino Acid	Mw	pI	Hydropathicity	Membrane Types
*TaWSD01-A*	*TraesCS1A02G385500*	Chr1A:554830347−554835264(+)	1	470	52.08	9.29	−0.13	Transmembrane
*TaWSD01-B*	*TraesCS1B02G411900*	Chr1B:637937998−637945870(+)	1	535	58.27	9.31	−0.16	Transmembrane
*TaWSD01-D*	*TraesCS1D02G393500*	Chr1D:462991889−462997985(+)	1	538	58.3	9.3	−0.13	Transmembrane
*TaWSD02-B*	*TraesCS1B02G402900*	Chr1B:632654884−632660030(−)	2	509	56.14	7.79	0.04	Transmembrane
*TaWSD03-D*	*TraesCS1D02G029300*	Chr1D:11524330−11527811(+)	1	510	56.38	9.36	−0.09	Transmembrane
*TaWSD04-B*	*TraesCS2B02G007200*	Chr2B:4502931−4515467(+)	1	509	56.65	8.16	−0.1	Soluble
*TaWSD04L-B*	*TraesCS2B02G019100*	Chr2B:8766184−8773006(−)	1	512	57.07	8.35	−0.08	Transmembrane
*TaWSD04-D*	*TraesCS2D02G016300*	Chr2D:7876792−7889878(+)	1	515	57.54	8.74	−0.11	Soluble
*TaWSD05-D*	*TraesCS2D02G002200*	Chr2D:1912608−1922943(−)	1	496	55.43	9.67	−0.03	Soluble
*TaWSD06L-A*	*TraesCS3A02G013600*	Chr3A:9810278−9814698(−)	1	538	59.96	7.06	−0.02	Transmembrane
*TaWSD06-A*	*TraesCS3A02G013700*	Chr3A:9843405−9846523(−)	3	496	55.19	9.07	0.02	Transmembrane
*TaWSD06-B*	*TraesCS3B02G016400*	Chr3B:6670118−6673010(−)	3	500	55.68	9.07	0	Soluble
*TaWSD06-D*	*TraesCS3D02G012000*	Chr3D:4179986−4182963(−)	3	497	55.48	9.16	−0.02	Transmembrane
*TaWSD07-A*	*TraesCS3A02G017400*	Chr3A:11023281−11028199(−)	3	502	56.15	9.04	−0.01	Transmembrane
*TaWSD07-B*	*TraesCS3B02G029900*	Chr3B:13744964−13749320(+)	1	504	56.45	8.87	0.02	Transmembrane
*TaWSD08-A*	*TraesCS3A02G266700*	Chr3A:491222820−491226810(+)	1	518	57.76	7.75	−0.04	Transmembrane
*TaWSD08-B*	*TraesCS3B02G300200*	Chr3B:481649743−481652877(+)	1	509	56.58	8.77	0.02	Transmembrane
*TaWSD08-D*	*TraesCS3D02G266900*	Chr3D:370123379−370126365(+)	1	432	48.08	6.54	−0.01	Transmembrane
*TaWSD09-A*	*TraesCS3A02G266800*	Chr3A:491454647−491459021(+)	2	516	57.08	8.43	−0.11	Transmembrane
*TaWSD09-B*	*TraesCS3B02G300300*	Chr3B:481909951−481914364(+)	1	515	57.03	9.14	−0.13	Transmembrane
*TaWSD09-D*	*TraesCS3D02G267000*	Chr3D:370533318−370541637(+)	2	514	56.76	7.4	−0.09	Transmembrane
*TaWSD10-A*	*TraesCS3A02G297400*	Chr3A:532315417−532320407(+)	2	498	55.36	8.52	−0.03	Transmembrane
*TaWSD10-B*	*TraesCS3B02G339300*	Chr3B:545201896−545206411(−)	3	498	55.22	8.98	−0.02	Transmembrane
*TaWSD10-D*	*TraesCS3D02G304900*	Chr3D:418782594−418787008(−)	2	497	55.41	8.28	−0.02	Transmembrane
*TaWSD11-B*	*TraesCS3B02G006700*	Chr3B:3601450−3607208(+)	3	501	55.62	9.1	0.04	Transmembrane
*TaWSD11L-B*	*TraesCS3B02G011800*	Chr3B:5230206−5234785(−)	3	534	59.26	9.92	−0.07	Transmembrane
*TaWSD12-A*	*TraesCS5A02G321800*	Chr5A:534463680−534467925(+)	1	529	58.5	7.83	−0.01	Transmembrane
*TaWSD12-D*	*TraesCS5D02G328800*	Chr5D:421159276−421163649(−)	1	519	57.29	7.28	−0.01	Transmembrane
*TaWSD12-Un*	*TraesCSU02G179300*	ChrUn:270056904−270061362(−)	1	519	57.29	7.28	−0.01	Transmembrane
*TaWSD13-A*	*TraesCS5A02G321900*	Chr5A:534569465−534573522(+)	2	509	55.56	6.68	0.04	Transmembrane
*TaWSD13-B*	*TraesCS5B02G322700*	Chr5B:507370761−507374395(+)	1	508	55.47	6.09	0.02	Transmembrane
*TaWSD13-D*	*TraesCS5D02G328600*	Chr5D:421084477−421090379(−)	2	509	55.54	6.49	0.05	Transmembrane
*TaWSD14-A*	*TraesCS5A02G322100*	Chr5A:534625497−534629014(+)	1	518	57.57	9.06	0.05	Transmembrane
*TaWSD14L-B*	*TraesCS5B02G322300*	Chr5B:507058471−507062451(+)	2	516	56.9	6.96	0.08	Transmembrane
*TaWSD14-B*	*TraesCS5B02G322800*	Chr5B:507584712−507588567(+)	3	513	56.76	9.1	0.01	Soluble
*TaWSD14-D*	*TraesCS5D02G328500*	Chr5D:420968589−420977820(−)	1	516	56.88	8.48	−0.04	Soluble
*TaWSD15-B*	*TraesCS5B02G322500*	Chr5B:507187491−507192457(+)	1	513	56.72	6.87	0.05	Transmembrane
*TaWSD15-D*	*TraesCS5D02G328700*	Chr5D:421133448−421138978(−)	2	517	57.12	7.83	0.01	Transmembrane
*TaWSD16-B*	*TraesCS5B02G495000*	Chr5B:662851150−662856702(−)	1	464	51.65	6.88	0.01	Transmembrane
*TaWSD17-D*	*TraesCS6D02G014800*	Chr6D:6156126−6159148(−)	1	382	42.24	8.93	0.01	Transmembrane
*TaWSD17-Un*	*TraesCSU02G029300*	ChrUn:28199371−28204814(+)	2	498	55.33	7.8	−0.03	Transmembrane
*TaWSD18-D*	*TraesCS7D02G029000*	Chr7D:14426236−14437067(+)	1	499	55.77	8.19	−0.06	Transmembrane
*TaWSD19-D*	*TraesCS7D02G111200*	Chr7D:66953256−66958978(+)	1	524	57.89	8.64	−0.03	Soluble

## Data Availability

The original contributions presented in this study are included in the article/Supplementary Materials. Further inquiries can be directed to the corresponding author.

## Supplementary Materials

The following supporting information can be downloaded at: https://www.mdpi.com/article/10.3390/genes17030353/s1, Figure S1. Chromosomal localization of wheat WSDs. Figure S2. Duplication types of TaWSD genes. Figure S3. Phylogenetic analysis of WSD genes in five plant species. Figure S4. Conserved motif 3 and the key catalytic site HHXXXDG. Figure S5. 3D structure of TaWSDs. Figure S6. Group assignment of 201 wheat RNA-seq samples. Figure S7. Heatmap of PCCs among TaWSDs and their potential regulatory factors. Figure S8. Distribution of putative TFBSs in the promoters of the corresponding TaWSD genes. Table S1. Information of 63 species used for WSD phylogenetic analysis. Table S2. List of 201 wheat RNA-seq datasets from the Sequence Read Archive (SRA). Table S3. Primers used in qPCR experiment. Table S4. Subcellular localization of TaWSDs predicted by DeepLoc-2. Table S5. Collinear gene pairs identified by syntenic analysis and gene duplication modes of TaWSDs. Table S6. WSD homologs identified in 63 species with clade assignments. Table S7. 35 TFs exhibiting strong positive correlations with TaWSD expression in 201 samples. Table S8. 27 candidate TFs, their best-hit homologous proteins and corresponding TFBS motifs from the JASPAR database. Table S9. Genes reported to regulate cuticle wax biosynthesis in plants. Table S10. Genes from selected TF families known to regulate lamina joint development and leaf angle in rice and maize.

## Abbreviations

The following abbreviations are used in this manuscript:

WSD	Wax synthase/diacylglycerol acyltransferase
LJ	Lamina joint
TF	Transcription factor
PCC	Pearson correlation coefficients
TFBS	Transcription factor binding site
kDa	kiloDalton
AS	Alternative Splicing
CDS	Coding Sequence
UTR	Untranslated Region
