# Supplementary material for: Genome-Wide Identification and Expression Analysis of the WSD Gene Family in Wheat
Source: Genes (Basel). 2026 Mar 23;17(3):353. doi: 10.3390/genes17030353 (PMC13025745; doi:10.3390/genes17030353)

Supplementary Materials

Figure S1. Chromosomal localization of wheat WSDs.

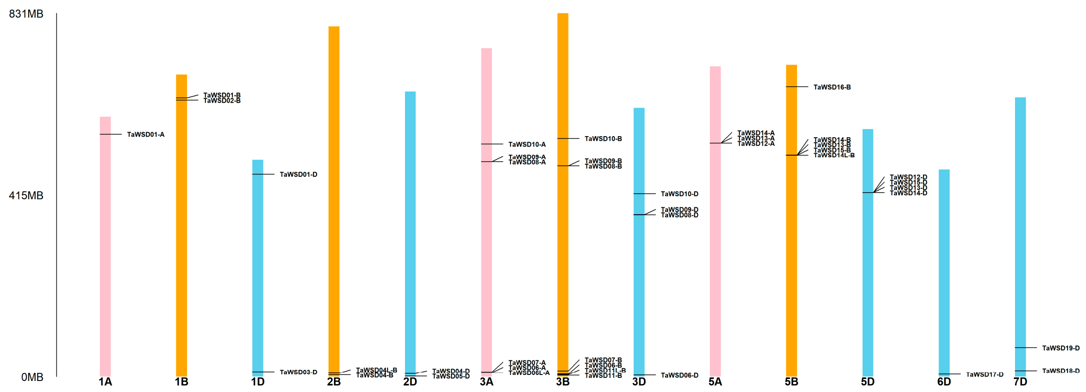

Figure S2. Duplication types of TaWSD genes.

A. Pie chart showing the proportions of different duplication types among TaWSD genes.

B. Presence (1) or absence (0) of syntenic orthologs for TaWSD duplication pairs in the progenitor species *T. turgidum*, and *A. tauschii*.

A B

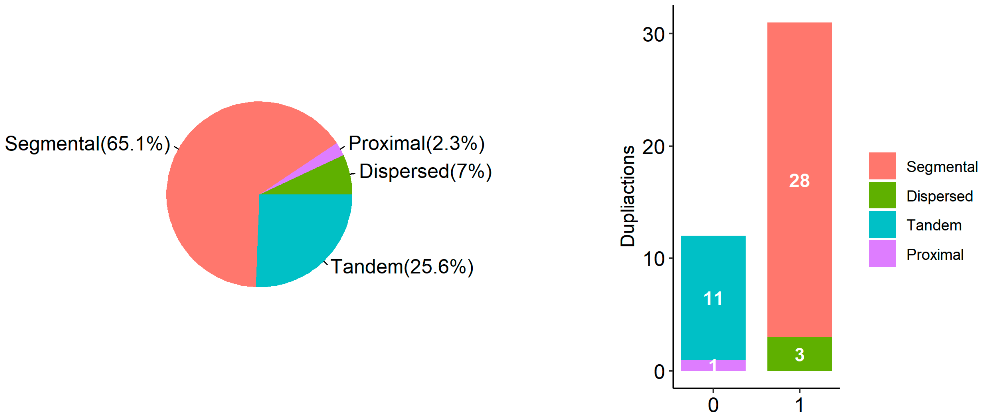

Figure S3. Phylogenetic analysis of WSD genes in five plant species.

Notes: The plant species includes *Triticum urartu* (tur), *Aegilops speltoides* (asp), rice (*Oryza sativa*, osa), maize (*Zea mays*, zma), and *Arabidopsis thaliana* (ath). The tree was rooted using a bacterial WSD homolog from *Acinetobacter sp.* as the outgroup.

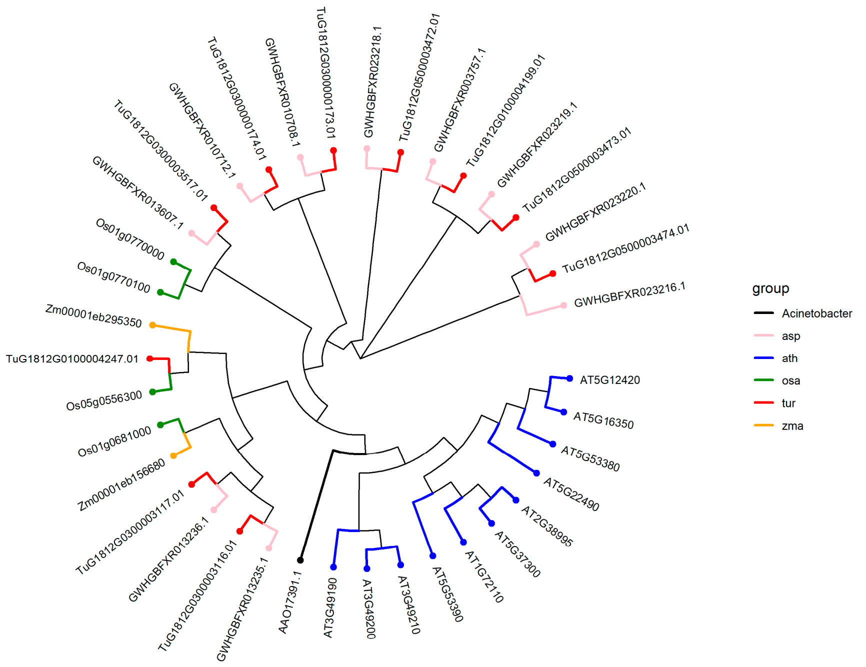

Figure S4. Conserved motif 3 and the key catalytic site HHXXXDG.

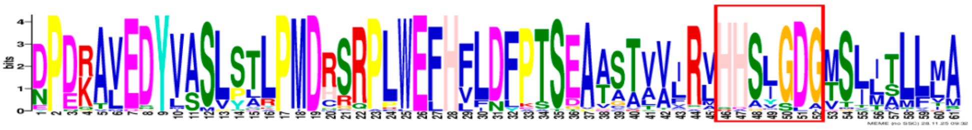

```
                         ....|....| ....|....| ....|....| ....|....| ....|....| ....|....| ....|....| ....|....| .
                                 10         20         30         40         50         60         70         80
TaWSD17-D N-terminus     -------MSV VSLSTQYMDN SRPLWDFHLL DFPTSE--AT STAVMRVHHS LSDGVSLLTL LMSCARSAAD PVKPPAMPPL P
TaWSD01-D motif M3       DPDRALEDYV SSLSTRPMDN SRPLWEFHVL DFPTSE--AA GAVAIRMHHS LGDGVSLISL LTA------- ---------- -
TaWSD01-B motif M3       DPDRALEDYV SSLSTRPMDH SRPLWEFHVI NLPTSE--AA GAVAIRMHHS LGDGVSLISL LTA------- ---------- -
TaWSD01-A motif M3       DPDRALEDYV SSLSTRPMDH SRPLWEFHVI DFPTSE--AA GAVAIRMHHS LGDGVSLISL LTA------- ---------- -
TaWSD09-D motif M3       DPDRALENYV SSLSTLPMDH SRPLWELHVL DFPTSD--AA AALVLRVHHS VGDGVSLLSL FIA------- ---------- -
TaWSD09-B motif M3       DPDRALEHYV SSLSTLPMDH SRPLWELHVF DFPTSD--AA AAVVLRVHHS VGDGVSLLSL FIA------- ---------- -
TaWSD09-A motif M3       DPDRALESYV SSLSTRPMDH SRPLWELHVL DFPTSD--AD AAVVLRVHHS VGDGVSLLSL FIA------- ---------- -
TaWSD08-A motif M3       EPDRTLEDYL SSLSTLPMDH SRPLWEFHVL DFPTSE--AA AALAFRAHHS LGDGTSLLSL LVA------- ---------- -
TaWSD08-B motif M3       EPNKTLEDYL SSLSTLPMDH SRPLWEFHVL DFPTSE--AV AALAFRAHHS LGDGTSLLSL LVA------- ---------- -
TaWSD08-D motif M3       EPDRTLEDYL SSLSTLPMDH SHPLWEFHVL DFPTSE--AV AALAFHAHHS LGDGTSLLSL LVA------- ---------- -
TaWSD17-Un motif M3      NPDQAVEDYV ASLPNKPMDH SRPLWEFHVL DFPTSE--AT ATVVMRVHHS LSDGTSLLML LLS------- ---------- -
TaWSD10-A motif M3       NPDKAVEDYV ASLSTLPMDR SQPLWEFHLF DFPTSE--AT STAAIRVHHS LGDGMSLLTL LMA------- ---------- -
TaWSD10-D motif M3       NPDKAVEDYV ASLSTLPMDR SQPLWEFHLF DFPTSE--AT STAAIRVHHS LGDGMSLLTL LMA------- ---------- -
TaWSD10-B motif M3       NPDKAVEDYV ASLSTLPMDR SQPLWEFHLF DFPTSE--AT STAAIRVHHS LGDGMSLLTL LMA------- ---------- -
TaWSD03-D motif M3       DPDKAVEDYV ASLSTAPMDR SRPLWELHFL NYPTTE--AA STVVIRVHHS LGDGMSLLTL LMA------- ---------- -
TaWSD06L-A motif M3      DPEKAVEDYV ASLSLLPMDM CRPFWEFHFL DFPTSE--AA STVVLRIHHS IGDGTSITTL LVA------- ---------- -
TaWSD06-A motif M3       DPEKAVEDYV ASLSLLPMDR RRPLWELHFL DFPSSE--AA STVVLRIHHS IGDGTSITTL LMA------- ---------- -
TaWSD06-D motif M3       DPEKAVEDYV ASLSLLPMDR RRPLWEFHFL DFPSSE--AA STVVLRIHHS IGDGTSITTL LMA------- ---------- -
TaWSD06-B motif M3       DPEKAVEDYV ASLSLLPMDR RRPLWEFHFL DFPSSE--AA STVVLRIHHS IGDGTSITTL LMA------- ---------- -
TaWSD18-D motif M3       NPEKAVEDYV ASLSTLPMDR RRPLWEFHFL NLETSE--AT STVVLRIHHS IGDAMSIMTI FMA------- ---------- -
TaWSD11L-B motif M3      NPEKAVEDYV AMLSTLPMDR GRPLWEFHFL NLKTSG--AA STVVLRIHHS IGDAMSIMTL FMA------- ---------- -
TaWSD11-B motif M3       NPEKAVEDYV AMLSTLPMDR GRPLWEFHFL NLKTSG--AA STVVLRIHHS IGDAMSIMTL FMA------- ---------- -
TaWSD05-D motif M3       DPDKAVEDYV SSLSTLPMDR SRPLWEFHFL DFATSE--AT STTVLRIHHS IGDGMSIMTL LMA------- ---------- -
TaWSD07-B motif M3       NPEKAMEDYV ASLSLLPMDR RRPLWEFHFL DFPTSE--AA STTVLRIHHS IGDGMSIMTL LMA------- ---------- -
TaWSD07-A motif M3       DPEKAVEDYV ASPSLLPMDR RRPLWEFHFL DFPTSE--AA STTVLRIHHS IGDGMSIITL LMA------- ---------- -
TaWSD19-D motif M3       YPDKAVEDYV ASLPTLPMDR SRALLEFHLL DFPTSE--AT STVAVRVHHA YGDGMSLMAL LMM------- ---------- -
TaWSD04-B motif M3       DPDKAVEDYI ASLSTLPMDR SRPPWEFHFL DFPTSE--AA STVAIRVHHA YGDGMSTMAL LMM------- ---------- -
TaWSD04-D motif M3       DPDKVVEDYV ASLSTLPIDR SRPPWEFHFL DFPTSE--AA STVVIRVHHA YGDGMSSMAL LMM------- ---------- -
TaWSD04L-B motif M3      DPDKAVEDYV ASLSTLPMDC SRPPWEFHFL DFPSSE--AA STVAIRVHHA YGDGMSTLAL LMM------- ---------- -
TaWSD16-B motif M3       NPDRAVEDYV ASLSVYPMDP SRPRWEFHLL DFPTSEPGVV STVAIRAHHS LGDGVSLMVL L--------- ---------- -
TaWSD15-D motif M3       DPDRAVEDYV ASVYTLPMDR SRPLWEFHFL DYPTSE--AA STVVFRVHHS LSDGMSLITM LLA------- ---------- -
TaWSD15-B motif M3       DPDRAVEDYV ASVYTLPMDC SRPLWEFHFL DFPT------ STVVFRVHHS LSDGMSLITM LLASARS--- ---------- -
TaWSD12-A motif M3       DPDRAVEDYV ASVYTLPMDR SRPLWEFHFL DFPTSE--AA STVVLRVHHS LGDGMSLITL LLA------- ---------- -
TaWSD12-Un motif M3      DPDRAVEDYV ASVYTLPMDR SRPLWEFHFL DFPTSE--AA STVVARVHHS LGDGMSLITL LLA------- ---------- -
TaWSD12-D motif M3       DPDRAVEDYV ASVYTLPMDR SRPLWEFHFL DFPTSE--AA STVVARVHHS LGDGMSLITL LLA------- ---------- -
TaWSD14-A motif M3       DPDRALEDYV ASLYTLPMDR SRPLWEFHFL DFPTSE--AA STAVIRIHHS LGDGMSLITL LLA------- ---------- -
TaWSD14-D motif M3       DPDRAVEDYV ASLPALPMDC SRPLWEFHFL DFPTSE--AA ATAVIRVHHS LGDGMSLITM LLA------- ---------- -
TaWSD14-B motif M3       NPDRALEDYV ASLPALPMDC SRPLWEFHFL DFPTSE--AT STAVIRVHHS LGDGMSLITM LLA------- ---------- -
TaWSD14L-B motif M3      DPDRAVEDYV ASLQALPMDC SRPLWEFHFL DFPTSE--AT STVVFRVHHS LGDGMSLITL LLA------- ---------- -
TaWSD02-B motif M3       DPDQAVDDYV ASLPELPMDR SRPLWEFHFL DFPTSD--AT STVVIRVHHS LGDGTSLIML LLA------- ---------- -
TaWSD13-A motif M3       DPDRAVEDYL ASLPALPMDR SRPLWEFHFL DFPTSE--AT STAVIRVHHS LGDGTTLIAL LLA------- ---------- -
TaWSD13-B motif M3       DPDRAVEDYM ASLPALPMDR SRPLWEFHFL DFPASE--AT CTAVIRVHHS LGDGTTLIAL LLA------- ---------- -
TaWSD13-D motif M3       DPDGAVEDYL ASLPALPMDR SRPLWEFHFL DFPTSE--AT CTAVIRVHHS LGDGTTLIAL LLA------- ---------- -
```

Figure S5. 3D structure of TaWSDs.

Cyan represents α-helices, magenta represents β-sheets, salmon represents loops/random coils, and red represents the key motif HHXXXDG.

Clade06

TaWSD01-A

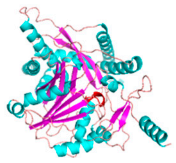

TaWSD01-B

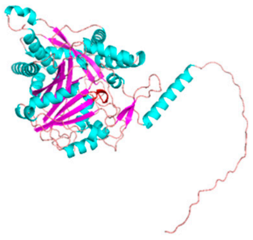

TaWSD01-D

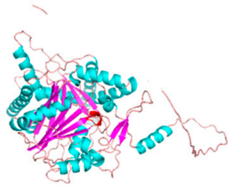

TaWSD09-A

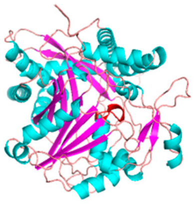

TaWSD09-B

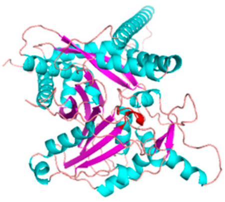

TaWSD09-D

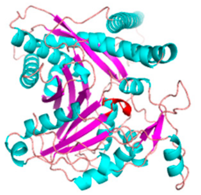

TaWSD08-A

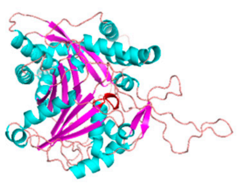

TaWSD08-B

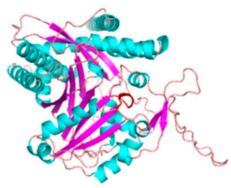

TaWSD08-D

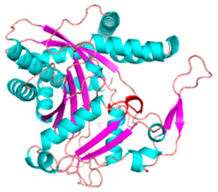

Clade09

TaWSD010-A

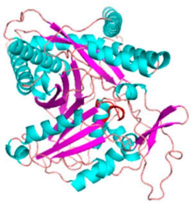

TaWSD010-B

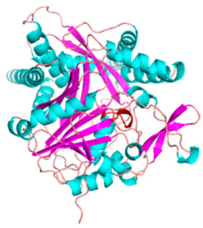

TaWSD010-D

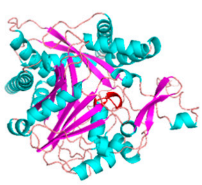

TaWSD017-D

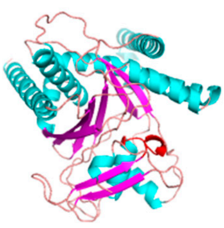

TaWSD017-Un

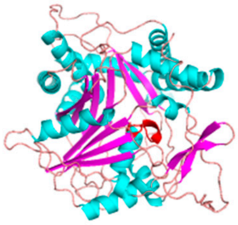

Clade10

TaWSD03-D

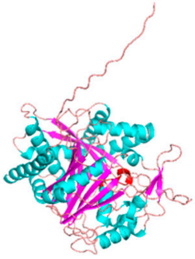

Clade12-Group A

TaWSD02-B

TaWSD04-B

TaWSD04-D

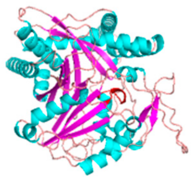

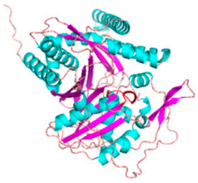

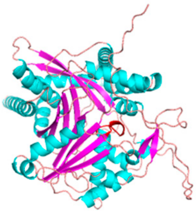

TaWSD04L-B

TaWSD12-A

TaWSD12-D

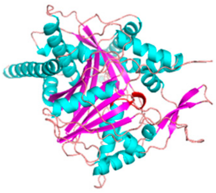

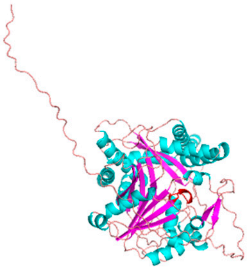

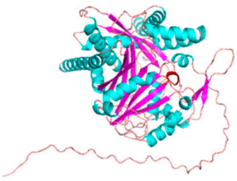

TaWSD12-Un

TaWSD13-A

TaWSD13-B

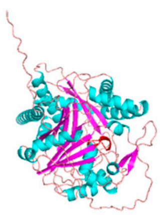

TaWSD13-D

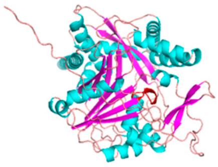

TaWSD14-A

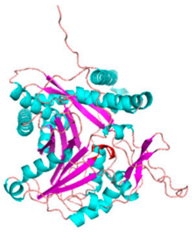

TaWSD14-B

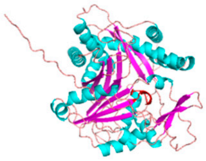

TaWSD14-D

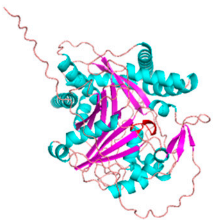

TaWSD14L-B

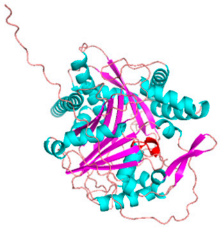

TaWSD15-B

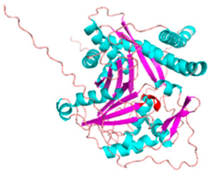

TaWSD15-D

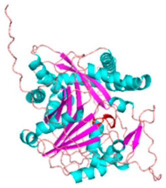

TaWSD16-B

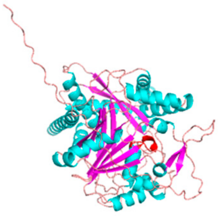

TaWSD19-D

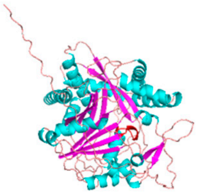

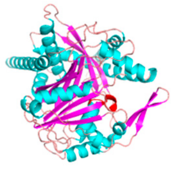

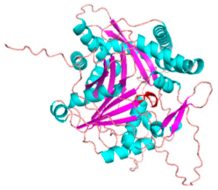

Clade12-Group B

TaWSD05-D

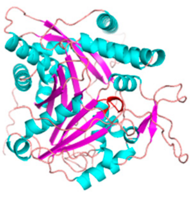

TaWSD06-A

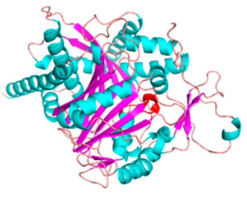

TaWSD06-B

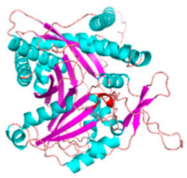

TaWSD06-D

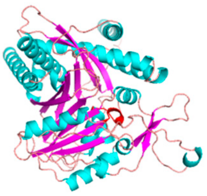

TaWSD06L-A

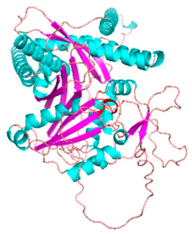

TaWSD07-A

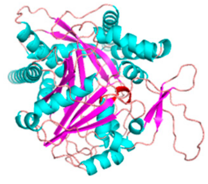

TaWSD07-B

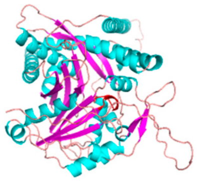

TaWSD11-B

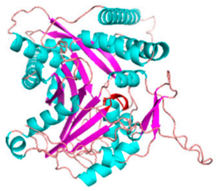

TaWSD11L-B

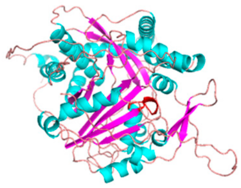

TaWSD18-D

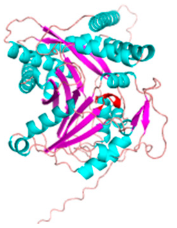

Figure S6. Group assignment of 201 wheat RNA-seq samples.

A. Clustering and group assignment of 201 wheat RNA-seq samples.

B. The PCA analysis within the tissue samples.

A

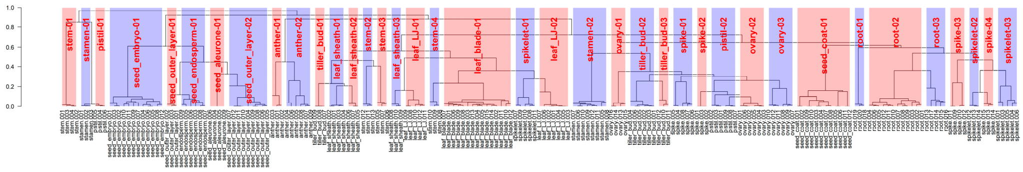

B

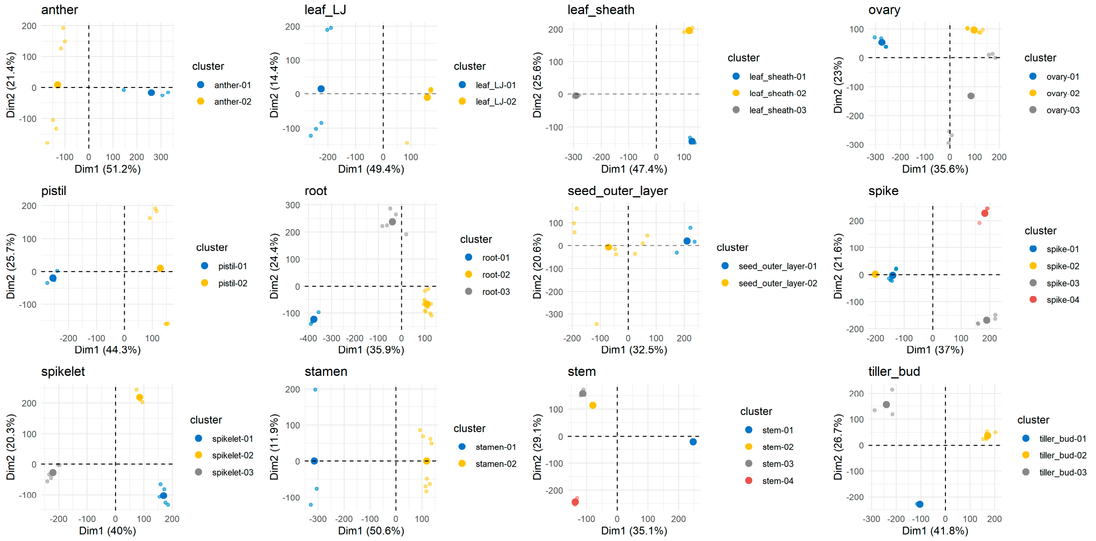

Figure S7. Heatmap of PCCs among TaWSDs and their potential regulatory factors.

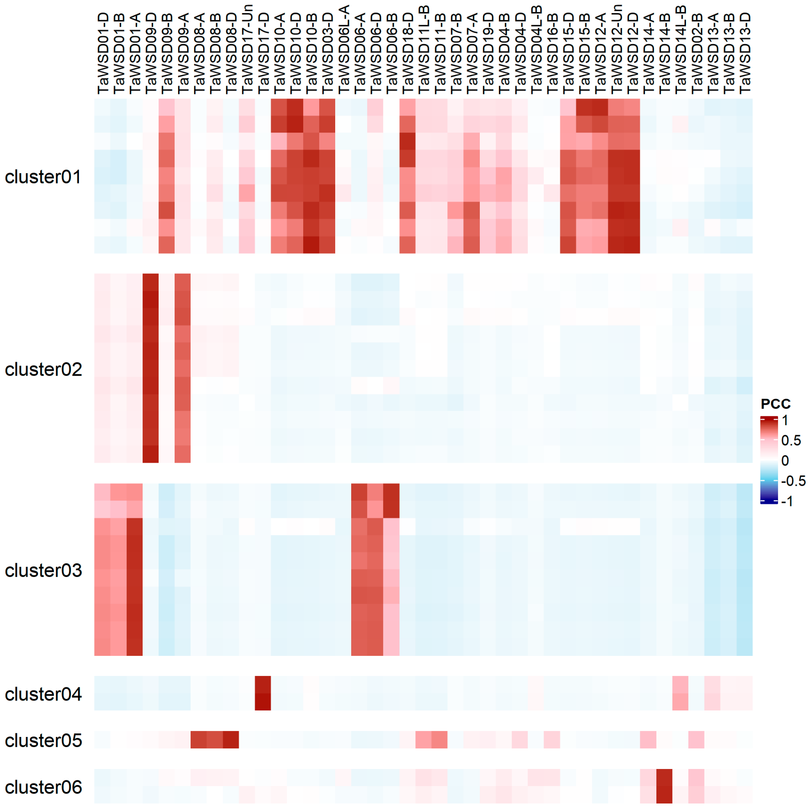

Figure S8. Distribution of putative TFBSs in the promoters of the corresponding TaWSD genes. The red square represent the TFBS-target pairs which were consistent with the predictions based on positive expression correlations.

	TaWSD03-D	TaWSD12-D	TaWSD12-Un	TaWSD13-B	TaWSD15-B	TaWSD17-D	TaWSD17-Un	TaWSD13-D	TaWSD01-A	TaWSD04-D	TaWSD09-A	TaWSD10-A	TaWSD10-B	TaWSD12-A	TaWSD14L-B	TaWSD01-B	TaWSD08-A	TaWSD08-B	TaWSD08-D	TaWSD14-B	TaWSD16-B	TaWSD05-D	TaWSD09-B	TaWSD13-A	TaWSD07-A	TaWSD07-B	TaWSD11L-B	TaWSD15-D	TaWSD02-B	TaWSD01-D	TaWSD10-D	TaWSD11-B	TaWSD14-D	TaWSD06L-A	TaWSD04L-B	TaWSD04-B	TaWSD19-D
MA0566.1	2	1	2	1	1	1	3	0	0	0	0	0	0	0	0	0	0	0	0	0	0	0	0	0	0	0	0	0	0	0	0	0	0	0	0	0	0
MA0569.2	0	1	2	0	1	0	0	1	0	0	0	0	0	0	0	0	0	0	0	0	0	0	0	0	0	0	0	0	0	0	0	0	0	0	0	0	0
MA0783.1	1	0	0	0	0	0	0	0	1	1	1	1	1	1	1	0	0	0	0	0	0	0	0	0	0	0	0	0	0	0	0	0	0	0	0	0	0
MA0959.1	2	0	1	1	0	1	3	0	0	0	1	0	0	0	0	1	3	3	3	2	1	0	0	0	0	0	0	0	0	0	0	0	0	0	0	0	0
MA1037.2	0	0	0	0	0	0	0	0	0	0	0	0	0	0	1	0	1	1	0	0	0	1	1	1	0	0	0	0	0	0	0	0	0	0	0	0	0
MA1199.1	0	0	0	0	0	0	0	0	0	0	0	0	0	1	0	0	0	1	0	0	0	0	1	0	1	1	1	1	0	0	0	0	0	0	0	0	0
MA1293.2	0	1	0	1	0	0	0	0	0	0	0	0	0	0	0	0	0	0	1	0	1	0	1	0	0	0	0	0	1	0	0	0	0	0	0	0	0
MA1296.2	0	0	0	0	0	0	0	0	0	0	0	0	1	0	0	0	0	0	0	0	0	0	0	0	1	0	1	0	0	1	1	1	1	0	0	0	0
MA1310.2	0	0	0	0	0	1	0	0	0	0	0	0	0	0	0	0	0	2	0	0	0	0	0	0	0	0	0	1	0	0	2	0	0	0	0	0	0
MA1314.2	0	0	1	0	0	0	0	0	0	1	0	0	0	0	0	0	0	1	0	0	0	0	0	0	0	0	0	0	0	0	0	0	0	0	0	0	0
MA1350.1	0	0	0	1	0	1	2	0	1	0	1	1	0	1	0	0	0	1	0	0	0	1	0	0	0	1	1	0	0	0	0	1	0	1	0	0	0
MA1363.1	0	1	1	0	0	0	0	0	0	1	2	0	0	1	0	0	0	0	1	0	1	1	0	0	0	0	0	0	0	0	0	0	0	0	1	0	0
MA1667.3	1	0	1	0	1	0	0	1	0	3	2	0	1	0	0	1	0	0	0	0	0	0	0	1	0	1	0	0	0	0	0	0	0	0	0	0	0
MA1823.2	0	0	0	2	0	0	0	0	1	0	0	0	0	1	0	1	0	0	0	0	0	0	0	1	2	0	0	1	0	1	2	0	1	1	0	1	1
MA1835.2	1	1	1	3	0	0	0	4	0	0	0	0	0	1	0	0	0	0	0	1	0	0	0	0	0	0	1	0	0	0	0	1	1	0	0	0	0

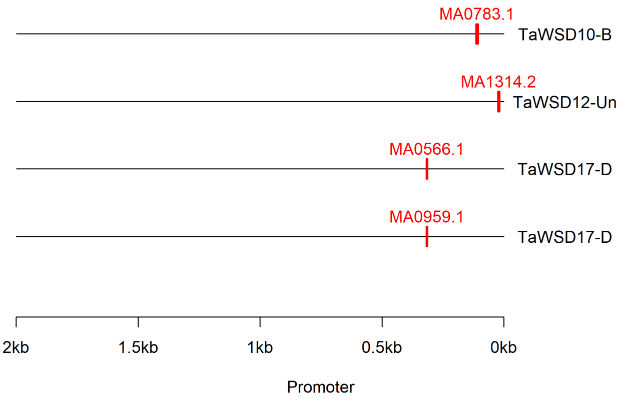

Supplement: Supplementary file 1 [file genes-17-00353-s001.zip › Supplementary Figures.pdf]
